# Risiko- und Komplikationsprofil orthogeriatrischer Patienten bei elektivem Hüft- und Kniegelenkersatz

**DOI:** 10.1007/s00391-024-02295-3

**Published:** 2024-04-19

**Authors:** Tobias Kappenschneider, Günther Maderbacher, Matthias Meyer, Stefano Pagano, Jan Reinhard, Katrin Michalk, Joachim Grifka, Dominik Emanuel Holzapfel

**Affiliations:** Klinik und Poliklinik für Orthopädie der Universität Regensburg, Asklepios Klinikum Bad Abbach, Kaiser-Karl V.-Allee 3, 93077 Bad Abbach, Deutschland

**Keywords:** Orthogeriatrie, Risikofaktoren, Hüft-TEP, Knie-TEP, Postoperative Komplikationen, Orthogeriatrics, Risk factors, THA, TKA, Postoperative complications

## Abstract

**Hintergrund:**

Die Versorgung geriatrischer Patienten bei elektiven orthopädischen Eingriffen gewinnt aufgrund der demografischen Entwicklung zunehmend an Bedeutung. Im Vergleich zur Alterstraumatologie existieren hierbei in Deutschland jedoch noch keine etablierten orthogeriatrischen Versorgungsmodelle und daher kaum wissenschaftliche Daten. Ziel dieser Studie war die Darstellung des Risiko- und Komplikationsprofils bei älteren Patienten mit elektivem Hüft- und Kniegelenkersatz.

**Methodik:**

Im Rahmen einer prospektiven Studie wurden Daten orthogeriatrischer Patienten mit Indikation zur elektiven Hüft- und Knietotalendoprothese nach Erfüllung definierter Ein- und Ausschlusskriterien im Zeitraum zwischen Januar 2021 und August 2023 in der Orthopädie eines deutschen Universitätsklinikums zur deskriptiven Analyse eines Risiko- und Komplikationsprofils erhoben. Neben einer prä- und perioperativen Datenanalyse erfolgten Nachbeobachtungen 4 bis 6 Wochen und 3 Monate postoperativ.

**Ergebnisse:**

Das operative Risikoprofil des untersuchten Patientenguts zeichnete sich durch hohes Alter (78,4 ± 4,8 Jahre), Prä‑/Adipositas (76 %), Multimorbidität (7,4 ± 3,1 Komorbiditäten), Polypharmazie (7,5 ± 3,8 Präparate), Immobilität (Short Physical Performance Battery 7,1 ± 2,6), Pre‑/Frailty (87 %), häufige Antikoagulation (22 %) und hohe Anzahl an potenziell inadäquater Medikation (64 %) aus. Komplikationsereignisse traten v. a. innerhalb der ersten 7 Tage postoperativ auf. Etwa 90 % der Ereignisse innerhalb dieses Erfassungszeitraumes beliefen sich auf „Minor“-Komplikationen. Im weiteren Verlauf sank die Gesamtkomplikationsrate deutlich.

**Schlussfolgerung:**

Aufgrund des hohen Risiko- und Komplikationsprofils sollte zukünftig der routinemäßige Einsatz orthogeriatrischer Co-Management-Modelle bei elektiven orthopädischen Eingriffen geprüft werden.

**Graphic abstract:**

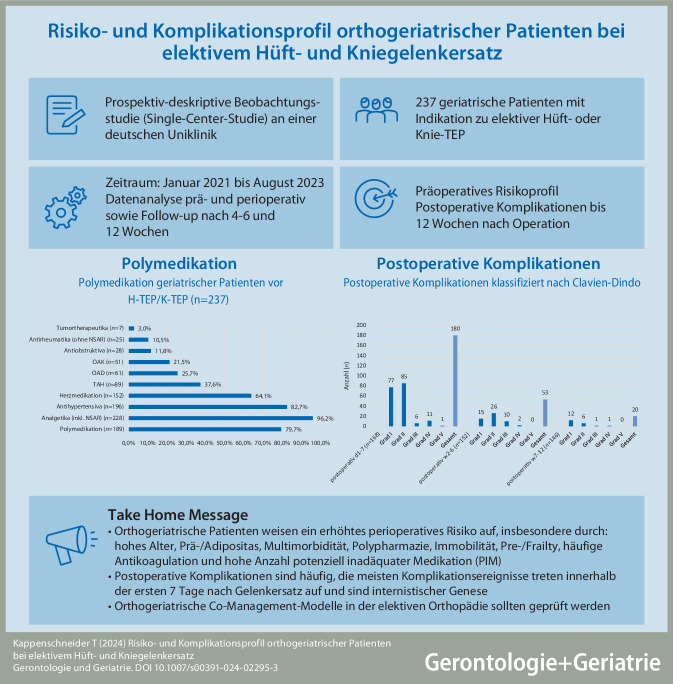

**Zusatzmaterial online:**

Zusätzliche Informationen sind in der Online-Version dieses Artikels (10.1007/s00391-024-02295-3) enthalten.

## Abstract (en)

**Background:**

The care of geriatric patients undergoing elective orthopedic surgery is becoming increasingly more important due to demographic trends. Compared to geriatric traumatology, however, there are still no established orthogeriatric care models in Germany and therefore hardly any scientific data. The aim of this study was to describe the risk and complication profiles in older patients with elective hip and knee replacements.

**Methods:**

In a prospective study data were collected from orthogeriatric patients with indications for elective hip and knee replacement surgery who fulfilled defined inclusion and exclusion criteria between January 2021 and August 2023 in the orthopedic department of a German university hospital for the descriptive analysis of risk and complication profiles. In addition to a preoperative and perioperative data analysis, a follow-up was conducted 4–6 weeks and 3 months postoperatively.

**Results:**

The surgical risk profile of the patient population analyzed was characterized by advanced age (78.4 ± 4.8 years), preobesity/obesity (76%), multimorbidity (7.4 ± 3.1 comorbidities), polypharmacy (7.5 ± 3.8 medications), immobility (short physical performance battery 7.1 ± 2.6), prefrailty/frailty (87%), frequent anticoagulation (22%) and a high number of potentially inappropriate medications (64%). Complication events mainly occurred within the first 7 days postoperatively and 90% of the events within this recording period were minor complications. The overall complication rate significantly decreased in the follow-up period.

**Conclusion:**

Due to the high risk and complication profiles the routine use of orthogeriatric co-management models for elective orthopedic surgery should be considered in the future.

Die Behandlung geriatrischer Patienten in der Orthopädie gewinnt aufgrund der demografischen Entwicklung weiter an Bedeutung. Vor allem in der Endoprothetik ist mit dramatisch steigenden Patientenzahlen zu rechnen. Doch die klinische Versorgung älterer Patienten in der elektiven Orthopädie weist im Vergleich zur Alterstraumatologie einige Unterschiede auf. Welche Anforderungen werden hierbei an den Geriater gestellt? Diese Originalarbeit soll einen Überblick über das Risiko- und Komplikationsprofil orthogeriatrischer Patienten bei elektivem Hüft- und Kniegelenkersatz geben.

Etwa die Hälfte der Weltbevölkerung im Alter von 65 Jahren und älter ist von einer Arthrose betroffen [1, 2]. Die Prävalenz der Osteoarthrose nimmt mit dem Alter unaufhaltsam zu, da die Erkrankung nicht reversibel ist. Cox- und Gonarthrosen sind Hauptursachen für eingeschränkte Mobilität, insbesondere bei älteren Menschen [3, 4]. Für sie stellen orthopädische Operationen oft die einzige Möglichkeit zur Wiedererlangung der Mobilität und Steigerung der Lebensqualität dar. So ist die Anzahl der Hüft- und Knietotalendoprothesen (H-TEP und K‑TEP) weltweit in den letzten 2 Jahrzehnten deutlich gestiegen [5, 6].

Trotz neuerer Operationstechniken haben ältere Patienten, insbesondere ≥ 80 Jahre, ein erhöhtes Komplikations- und Mortalitätsrisiko bei solchen Eingriffen [7]. Neben dem unabhängigen Risikofaktor Alter und der damit verbunden erhöhten Vulnerabilität zeichnen sich diese Patienten oftmals durch Multimorbidität, Immobilität, Polypharmazie und Frailty aus. Weiter sind Besonderheiten durch eine Niereninsuffizienz sowie Aspekte in der perioperativen Anwendung von Antikoagulanzien häufig zu beachten. Auch kognitive Störungen mit erhöhtem postoperativen Delirrisiko spielen eine Rolle.

Bisher noch zu wenig Beachtung wird den sog. modifizierbaren Risikofaktoren geschenkt. Hierzu zählen v. a. Anämie, Malnutrition, Adipositas, Nikotinabusus sowie ein insuffizient eingestellter Diabetes mellitus [8]. Ein weiterer vielversprechender Ansatz in der Risikostratifizierung scheint die Erfassung des Frailty-Syndroms zu sein. Zusammenfassend kann Frailty als mehrdimensionales geriatrisches Syndrom, das durch den Verlust individueller Reservekapazitäten und eine erhöhte Anfälligkeit für interne und externe Stressoren gekennzeichnet ist, beschrieben werden [9]. Ein Zusammenhang zwischen Frailty und schlechten Ergebnissen nach Gelenkersatz konnte bereits nachgewiesen werden. Gebrechliche Patienten zeigten 4‑ bis 6fach höhere Komplikationsraten nach Hüft- und Knie-TEP [10]. Auch in anderen Bereichen der Orthopädie, beispielsweise bei Wirbelsäuleneingriffen ist das Frailty-Syndrom Prädiktor für unerwünschte Ereignisse [11]. Modifizierbare Risikofaktoren können im Rahmen der Operationsvorbereitung erkannt und optimiert werden. Dadurch lässt sich das Operationsrisiko entscheidend verringern [8].

Hier unterscheidet sich der geriatrische Patient mit elektiver Operation in der Orthopädie vom alterstraumatologischen Patienten essenziell, bei welchem aus Zeitgründen eine präoperative Optimierung nur bedingt möglich ist. Jedoch wird diese „diagnostische Lücke“ im klinischen Alltag bisher kaum adressiert. Es existieren weder adäquates Screening zur Erkennung eines „geriatrischen Patienten“ für elektive orthopädische Eingriffe, noch findet eine präoperative geriatrische Mitbeurteilung oder gar ein präoperatives geriatrisches Assessment routinemäßig statt. Jedoch konnten bereits Harari et al. 2007 zeigen, dass genau jene präoperativen Aspekte in Kombination mit einem orthogeriatrischen Co-Management postoperativ zu einem deutlich besseren Outcome bei elektiven orthopädischen Eingriffen führen können. So wiesen diese Patienten signifikant weniger postoperative Komplikationen, ein deutlich besseres funktionelles Ergebnis sowie eine um mehrere Tage verkürzte Hospitalisierungsdauer auf [12]. Um diese Versorgungslücke in Deutschland zu schließen, wird seit 2021 im Rahmen des Modellprojekts „Spezielle Orthopädische Geriatrie (SOG)“ des Gemeinsamen Bundesausschusses (G-BA) ein neues, auf die orthopädische Elektivsituation ausgerichtetes Versorgungskonzept, welches im Erfolgsfall eine neue Behandlungsprozedur ähnlich zur geriatrischen frührehabilitativen Komplexbehandlung (GFK) in der Alterstraumatologie abbilden könnte, wissenschaftlich evaluiert [13].

Ziel dieser Studie war es, einerseits das Risikoprofil orthogeriatrischer Patienten vor Hüft- und Knie-TEP genauer zu untersuchen, anderseits aber auch einen umfassenden Überblick über die postoperativen Komplikationen innerhalb der ersten 3 Monate nach entsprechendem Gelenkersatz zu erhalten.

## Methodik

Zwischen Januar 2021 und August 2023 wurde in der orthopädischen Hochschulambulanz der Universität Regensburg bei insgesamt 237 Patienten im Rahmen der Indikationsstellung zur elektiven Hüft- oder Knie-TEP anhand von Vorbefunden, Medikamentenplänen, Eigen- und Fremdanamnese, telefonischem Kontakt mit behandelnden Haus- und Fachärzten, klinischer Untersuchung, Laboruntersuchung und geriatrischem Assessment (Tab. 1) ein umfassendes präoperatives Risikoprofil erstellt. Polymedikation wurde definiert als ≥ 5 Präparate. Hierbei wurden alle systemisch wirksamen Medikamente, inklusive β‑Blocker-haltige Augentropfen und Inhalativa berücksichtigt. Grundlage für die Einschätzung des Vorliegens einer potenziell inadäquaten Medikation (PIM) waren die PRISCUS (2010)- und FORTA-Listen [14, 15].

**Tab. 1:** Baseline-Daten, präoperativ

	**Orthogeriatrisches Patientenkollektiv (*n*** **=** **237)**
Alter in Jahren	78,4 ± 4,8 (70–89)
Weibliches Geschlecht *n* (%)	153 (65 %)
Präadipositas (BMI 25–29,9 kg/m^2^)	86 (36,3 %)
Adipositas (BMI ≥ 30 kg/m^2^)	94 (39,7 %)
Adipositas Grad I (BMI 30–34,9 kg/m^2^)	66 (27,8 %)
Adipositas Grad II (BMI 35–39,9 kg/m^2^)	21 (8,9 %)
Adipositas Grad III (BMI > 39,9 kg/m^2^)	7 (3,0 %)
Anzahl der Neben‑/Begleiterkrankungen	7,4 ± 3,1 (2–22)
Anzahl der Medikamente	7,5 ± 3,8 (0–21)
	**Orthogeriatrisches Patientenkollektiv (*n*** **=** **173)**
Ganggeschwindigkeit V in m/s	0,8 ± 0,3 (0,1–1,6)
Short Physical Performance Battery (SPPB) (0–12)	7,1 ± 2,6 (1–12)
*Frailty-Kriterien nach Fried (0–5)*	2,1 ± 1,3 (0–4)
Robust (0)	23 (13,3 %)
Pre-Frail (1–2)	74 (42,7 %)
Frail (≥ 3)	76 (44,0 %)

Angaben in Mittelwert ± Standardabweichung (Reichweite) oder Anzahl *n* (%), *BMI:* Body Mass Index

Als Einschlusskriterien wurden primäre Cox- oder Gonarthrose, Alter ≥ 70 Jahre + geriatrietypische Multimorbidität oder Alter ≥ 80 Jahre mit Indikation für elektiven Hüft- oder Kniegelenkersatz definiert. Ausschlusskriterien waren Alter < 70 Jahre, vorherige Fraktur/knöcherne Operation oder Tumor im Bereich des zu behandelnden Gelenks, akute Infektion und Pflegegrad ≥ 4.

Von den 237 rekrutierten Patienten stellten sich im oben genannten Zeitraum 173 Patienten in der zentralen Patientenaufnahme (ZPA) zur Operation vor. Die übrigen Patienten entschieden sich gegen eine Operation, ließen sich an einer anderen Klinik operieren, konnten aufgrund der Coronapandemie im Studienzeitraum nicht operiert werden, benötigten vorab umfassendere medizinische Diagnostik oder Behandlungen, lehnten die Teilnahme an der Studie ab oder waren zwischenzeitlich verstorben. In der ZPA erfolgten eine erneute Laboruntersuchung sowie weiterführendes geriatrisches Assessment (Tab. 1).

Von den 173 Patienten konnten 168 Patienten operiert und somit zur Erfassung des Komplikationsprofils berücksichtigt werden. Bei 5 Patienten musste aufgrund fehlender Operationsfähigkeit nach präoperativer Vorstellung in der ZPA die geplante Operation abgesetzt werden. Der stationäre Aufenthalt betrug in der Regel 7 Tage, in denen täglich eine Komplikationserfassung nach Clavien-Dindo [16] erfolgte. Zu einer Verlängerung der Verweildauer kam es lediglich aufgrund medizinscher Notwendigkeit oder Wartezeit bis zur Reha. Nach 4 bis 6 Wochen und nach 3 Monaten wurde der Patient zur Nachbeobachtung nochmals in die Hochschulambulanz einbestellt, wobei die Erfassung von Komplikationen des weiteren Verlaufs erfolgte. Zum Follow-up 1 erschienen 16 Patienten nicht, beim Follow-up 2 gingen 22 Patienten verloren. Darüber hinaus abweichende Fallzahlen sind in den jeweiligen Tabellen/Abbildungen separat aufgeführt.

### Komplikationserfassung nach Clavien-Dindo

Sie dient der Erfassung und Kategorisierung postoperativer Komplikationen. Es existieren je nach erforderlicher Therapieform 5 Schweregrade [16]:

- Grad I: Abweichung vom normalen postoperativen Verlauf mit definierten Behandlungsregimen,
- Grad II: anderweitige Therapeutika/Bluttransfusionen,
- Grad III: Einsatz von chirurgischen, endoskopischen oder radiologischen Interventionen,
- Grad IV: lebensbedrohliche Komplikationen mit intensivmedizinischer Behandlung,
- Grad V: Tod.

Die geriatrischen Assessments Short Physical Performance Battery (SPPB) und Frailty Kriterien nach Fried sind im Zusatzmaterial online: e1 beschrieben.

### Statistik

Falls nicht anders erwähnt, wurden für die deskriptive Statistik die quantitativen Variablen als Mittelwerte ± Standardabweichung und die qualitativen Daten als absolute und relative Häufigkeiten angegeben. Die mathematisch-statistische Erfassung und Analyse erfolgte mittels Microsoft Excel (Version 2020, One Microsoft Way, Redmond, WA, USA).

## Ergebnisse

Zunächst gibt die Tab. 1 einen Überblick über die präoperativen Baseline-Daten des orthogeriatrischen Patientenguts.

**Abb. 1:**
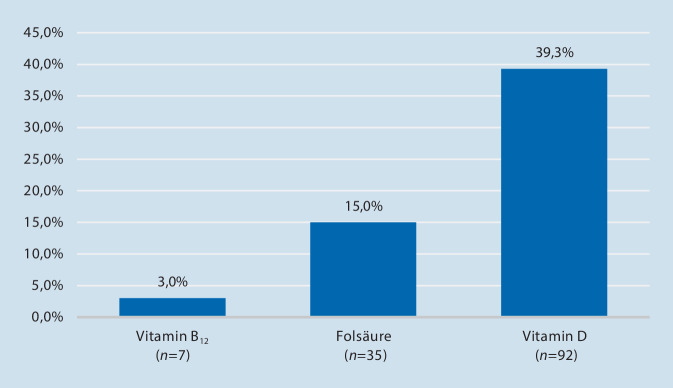
Präoperatives Screening auf Hypovitaminosen bei 234 Patienten (Mangelzustände wurden wie folgt definiert: Vitamin B_12_ < 200 pg/ml, Folsäure < 4,4 ng/ml, Vitamin D < 20 ng/ml)

In einem präoperativen Screening auf Vitaminmangelzustände konnte bei 3 % der Patienten ein Vitamin‑B_12_-Mangel, bei 15 % ein Folsäuremangel und bei ca. 39 % ein Vitamin-D-Mangel festgestellt werden (Abb. 1).

**Abb. 2:**
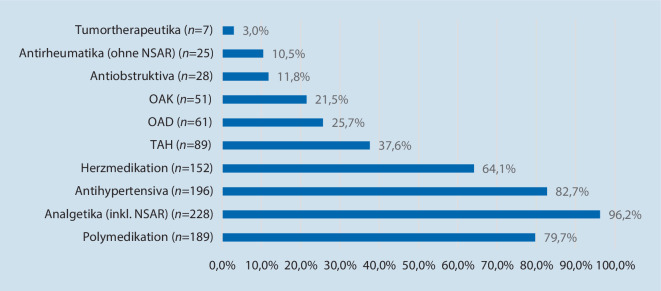
Häufigkeit ausgewählter Arzneimittel orthogeriatrischer Patienten (*n* = 237) bei H‑TEP/K-TEP. *NSAR* nichtsteroidale Antirheumatika, *OAK* orale Antikoagulanzien, *OAD* orale Antidiabetika, *TAH* Thrombozytenaggregationshemmer, Polymedikation definiert ≥ 5 Präparate

Nahezu 80 % der untersuchten Patienten wiesen bei Aufnahme zur Operation eine Polymedikation (≥ 5 Präparate) auf. Im Durchschnitt wurden 7,5 ± 3,8 verschiedene Medikamente eingenommen (0 bis 21 Präparate). Mit ca. 96 % nahmen fast alle Patienten mindestens ein Analgetikum (inklusive nichtsteroidale Antirheumatika) ein. Knapp 38 % benötigten Thrombozytenaggregationshemmer, und etwa jeder 5. Patient hatte eine orale Antikoagulation (Abb. 2).

**Abb. 3:**
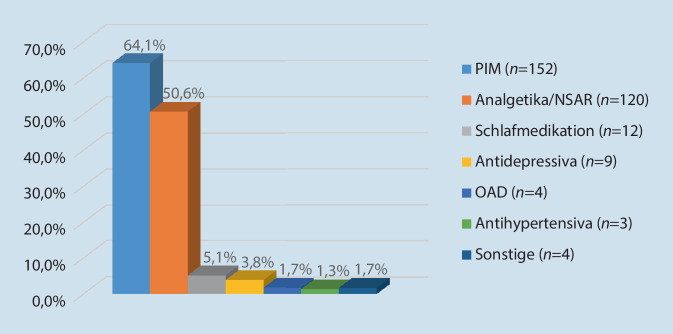
Potenziell inadäquate präoperative Medikation der 237 Patienten. *NSAR* nichtsteroidale Antirheumatika, *OAD* orale Antidiabetika

Bei Erstvorstellung in unserer Hochschulambulanz konnte bei Überprüfung der häuslichen Medikation bei ca. 64 % der Patienten eine potenziell inadäquate Medikation (PIM) festgestellt werden. 86 % davon wiesen hierbei ein PIM in der Dauermedikation auf, 11 % zwei PIM und 3 % sogar 3 verschiedene PIM. Mit Abstand am häufigsten wurden nichtsteroidale Antirheumatika (NSAR) (ca. 51 %) zur Langzeittherapie eingenommen, obwohl dies aufgrund von Alter und Komorbiditäten gemäß aktuellen Empfehlungen (PRISCUS-Liste/FORTA-Klassifikation) als potenziell inadäquat erachtet werden kann (Abb. 3).

**Abb. 4:**
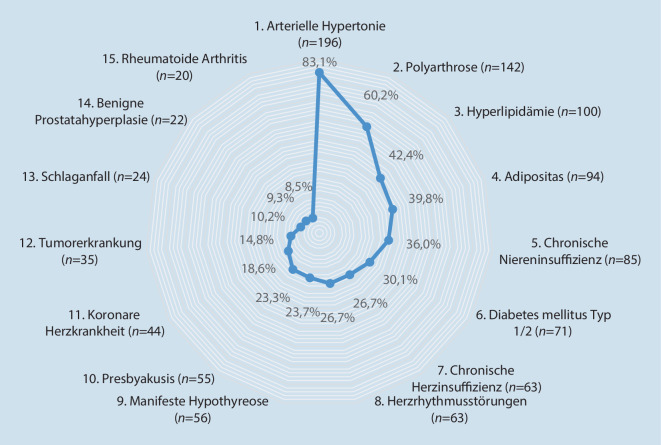
Die 15 häufigsten Komorbiditäten bei elektiver H‑TEP/K-TEP (*n* = 236)

Wie der Tab. 1 zu entnehmen ist, präsentierte das untersuchte orthogeriatrische Patientengut im Mittel 7,4 ± 3,1 Begleiterkrankungen. Über 83 % der Patienten, die sich zur elektiven Hüft‑/Knie-TEP vorstellten, litten an einer arteriellen Hypertonie. Mit Hyperlipidämie, Adipositas und Diabetes mellitus zählten 3 weitere kardiovaskuläre Risikofaktoren zu den häufigsten Komorbiditäten der TEP-Patienten. Mehr als jeder 3. Patient präsentierte eine chronische Niereninsuffizienz (Abb. 4).

**Abb. 5:**
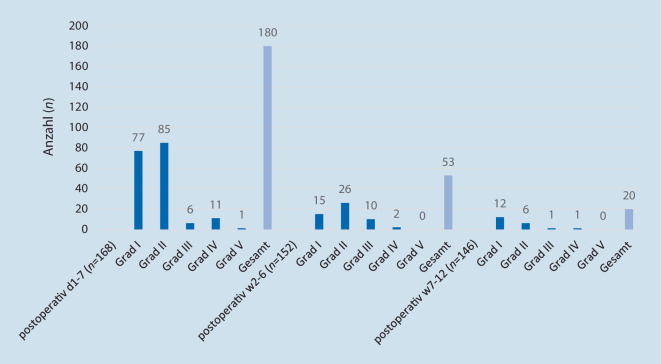
Postoperative Komplikationen klassifiziert nach Clavien-Dindo zu den verschiedenen Erfassungszeitpunkten nach H‑TEP/K-TEP

Rein quantitativ betrachtet, traten mit Abstand die meisten Komplikationsereignisse im direkten postoperativen Verlauf, innerhalb der ersten 7 Tage nach Gelenkersatz auf. 90 % der Ereignisse innerhalb dieses Erfassungszeitraumes beliefen sich auf „Minor“-Komplikationen (Clavien-Dindo I° und II°). 10 % waren „Major“-Komplikationen (Clavien-Dindo III° bis V°) mit chirurgischem, endoskopischem oder radiologischem Interventionsbedarf, intensivmedizinischer Behandlungsnotwendigkeit oder letalem Ausgang. Innerhalb der beiden weiteren Beobachtungszeiträumen (2. bis 6. Woche postoperativ und 7. bis 12. Woche postoperativ) sank die Komplikationsrate deutlich. Jedoch traten in der 2. bis 6. Woche nach TEP-Implantation weitere 12 „Major“-Komplikationen (Clavien-Dindo III° und IV°) auf, was einer relativen Häufigkeit von 8 % entspricht (Abb. 5).

**Abb. 6:**
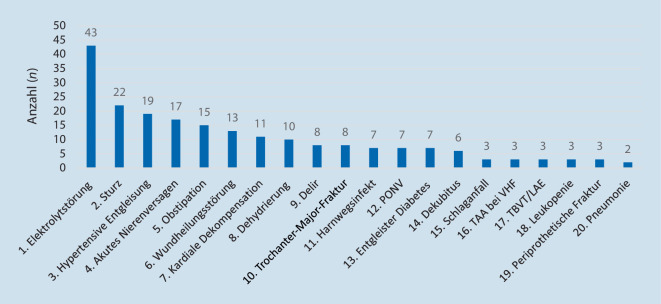
Überblick über die 20 häufigsten chirurgischen und nichtchirurgischen Komplikationen innerhalb der ersten 12 Wochen von 168 geriatrischen Patienten nach Operation. *PONV* „postoperative nausea and vomiting“, *TAA bei VHF* Tachyarrhythmia absoluta bei Vorhofflimmern, *TBVT/LAE* tiefe Beinvenenthrombose/Lungenarterienembolie

Wie Abb. 6 beschreibt, handelte es sich bei den meisten Komplikationsereignissen um Elektrolytstörungen (*n* = 43). Hierunter fielen eine Hypo‑/Hyperkaliämie sowie eine Hyponatriämie mit entsprechendem Korrekturbedarf. In 22 Fällen kam es im postoperativen Verlauf zu Stürzen, wobei sich 3 operierte Patienten eine periprothetische Fraktur zuzogen. Bei 8 Patienten trat ein postoperatives Delir auf. Die Delirrate lag somit bei 5 %.

## Diskussion

### Risikoprofil

Das Patientengut zeigt mit im Mittel 7,4 Begleiterkrankungen erwartungsgemäß ein hohes Maß an Multimorbidität. Dies deckt sich mit Angaben aus der Alterstraumatologie [17]. Das ebenfalls im Einklang mit bisheriger Literatur stehende hohe kardiovaskuläre Risikoprofil lässt sich sehr wahrscheinlich neben dem Alter an sich auch auf den durch die Arthrose bedingten Bewegungsmangel zurückführen [17, 18]. Die Mobilitätseinschränkung wird zudem in der reduzierten Ganggeschwindigkeit und dem erheblich verminderten SPPB-Score deutlich. In 76 % der Fälle lag in unserer Studie eine Prä‑/Adipositas vor. Eine dadurch erhöhte Rate an Wundheilungsstörungen, TEP-Infektionen, Thromboembolien und technischen Erschwernissen für den Operateur mit dadurch längeren Operationszeiten [8, 19] schärft das Risikoprofil orthogeriatrischer Patienten weiter. Ebenso präsentierten sich fast 87 % der Patienten in einem vor- oder sogar gebrechlichen Status. Dies steht im Einklang mit den Ergebnissen des *E*uropean *P*roject on *Os*teo*a*rthritis (EPOSA) von 2015, die eine klare Assoziation von Arthrose mit Frailty zeigen konnten [20]. Demgegenüber steht ohne operative Therapieoption der konservativ ausbehandelten Cox- oder Gonarthrose ein hohes Risiko für eine weitere Progredienz von Immobilität, Sarkopenie, Frailty, Morbidität sowie Verlust an Lebensqualität und Alltagskompetenz [3, 4].

Besonderer Bedeutung kommt dem perioperativen Management von Antikoagulanzien zu. Da mehr als jeder 5. Patient therapeutisch antikoaguliert war, spielt dies eine wichtige Rolle für den Geriater. Besorgniserregend ist mit ca. 64 % der hohe Anteil an potenziell inadäquater Medikation. Dies übertrifft sogar den Anteil an PIM von Thürmann et al., die 2022 in einer großen Querschnittanalyse von AOK-Versicherten einen PIM-Anteil bis zu 55 % eruierten [21]. Besonders auffällig ist in der untersuchten Kohorte der hohe Anteil an NSAR als Dauermedikation. Entgegen allgemeinen altersbedingten Empfehlungen [14, 15] und trotz Kontraindikationen wie kardialen Vorerkrankungen, Niereninsuffizienz oder gastrointestinalen Komorbiditäten nahm mehr als jeder Zweite präoperativ ein NSAR ein.

### Komplikationsprofil

Eine hohe Vulnerabilität geriatrischer Patienten für postoperative Komplikationen ist aus der Alterstraumatologie bekannt. So traten bei Darwich et al. 2021 in der Versorgung hüftgelenknaher Frakturen an einem regionalen Traumazentrum in 42 % der Fälle allgemeine und in 12 % der Fälle spezifisch-chirurgische Komplikationen auf [18]. In der elektiven Hüftendoprothetik konnten Boniello et al. in ihrer Arbeit von 2018 eine signifikant höhere Komplikationsrate und Mortalitätsrate für Patienten über dem 80. Lebensjahr nachweisen [7]. Im Einklang mit der beschriebenen Literatur zeigte sich auch in unserer Studie eine hohe Gesamtkomplikationsrate des untersuchten Patientenkollektivs. Als besonders vulnerable Phase bezüglich des Auftretens von Komplikationen zeichneten sich die ersten 7 Tage postoperativ bzw. die Tage des Indexaufenthalts ab. Im Gegensatz zu Boniello et al. erstreckt sich der Untersuchungszeitraum unserer Studie nicht nur retrospektiv auf 30 Tage nach der Operation, sondern inkludiert prospektiv die ersten 12 Wochen nach dem Gelenkersatz. Allerdings ist mit zunehmendem zeitlichem Abstand ein kausaler Zusammenhang zwischen der Operation und dem unerwünschten Ereignis auch kritischer zu betrachten. Dass die Anzahl der Gesamtkomplikationen des Indexaufenthalts die Gesamtzahl der operierten Patienten übersteigt, ist der Tatsache geschuldet, dass einzelne Patienten teils mehrere, verschiedene Komplikationsereignisse aufwiesen. Im Gegensatz zu einigen bisherigen Studien [7, 18] erfolgte die Komplikationserfassung nicht retrospektiv durch Auswertungen von Datenbanken, indem eben auch nur solche Daten ausgewertet werden konnten, die darin erfasst worden sind, sondern prospektiv durch eine täglich strukturierte Erfassung nach Clavien-Dindo [16]. Dies ermöglichte auch eine fachspezifische und somit sehr exakte Komplikationserfassung. So wurden chirurgische Ereignisse durch den Orthopäden und nichtchirurgische Komplikationen durch Internisten/Geriater registriert. Meyer et al. zeigten in ihrer Arbeit von 2020 einen eindeutigen Zusammenhang zwischen Frailty und postoperativen Komplikationen nach Hüft- und Knie-TEP [10]. In Anbetracht des hohen Anteils an Patienten mit Pre‑/Frailty unseres Patientenkollektivs ist die Anzahl der erfassten postoperativen Komplikationen nachvollziehbar. Analog zu Darwich et al. traten mit Abstand am meisten internistische Ereignisse auf [18]. Neben Elektrolytstörungen kam es auch häufiger zu Blutdruckentgleisungen und akuten Nierenversagen. Im Kontext von beiden Letzteren sollte der regelhafte Einsatz von NSAR in der Orthopädie bei geriatrischen Patienten kritischer evaluiert und mögliche Zusammenhänge sollten hierbei studienbasiert überprüft werden.

Erste, kürzlich veröffentlichte Zwischenergebnisse der noch laufenden SOG-Studie deuten auf eine erhebliche Risikoreduktion postoperativer Komplikationen bei geriatrischen Patienten nach elektiver Hüft- und Knie-TEP-Implantation durch ein orthogeriatrisches Co-Management hin [22]. Hierbei wird im Rahmen einer randomisierten kontrollierten Studie der Effekt eines orthogeriatrischen Co-Management-Modells, bestehend aus den 5 in sich ineinandergreifende Komponenten Screening, umfassendes präoperatives geriatrisches Assessment, präoperative Intervention, Fast-Track-Prinzip und multimodale perioperative Versorgung im orthogeriatrischen Team im Vergleich zur orthopädischen Standardversorgung bei geriatrischen Patienten in der elektiven, primären Hüft- und Kniegelenkendoprothetik untersucht [13]. Die Endergebnisse dieser deutschen Studie werden 2025 erwartet.

### Limitationen

Die Arbeit bildet einen Querschnitt der aktuellen Versorgung elektiver H‑TEP/K-TEP-Patienten in der geriatrischen Orthopädie ab. Es erfolgt keine Differenzierung zwischen einer Versorgung mit und ohne geriatrischem Co-Management. Die Daten wurden an einem zertifizierten Endoprothetikzentrum der Maximalversorgung erhoben, mit einerseits hoher Anzahl endoprothetischer Eingriffe pro Jahr, andererseits aber auch komplizierten Fällen und morbiden Patienten. Es handelt sich hierbei um eine „Single-Center“-Studie mit dementsprechenden Limitationen in Umfang und Heterogenität der Studienpopulation sowie möglichem „Zentrumsbias“.

## Ausblick

Orthogeriatrische Patienten profitieren unzweifelhaft von einem Hüft- und Kniegelenkersatz.

Jedoch müssen die altersbedingt erhöhte Vulnerabilität, das Risikoprofil und die erhöhte Komplikationsrate ebenso berücksichtigt werden. Beides gelingt unserer Ansicht nach nur durch eine integrierte Geriatrie in der Orthopädie ähnlich zur Alterstraumatologie.

## Fazit für die Praxis

- Orthogeriatrische Patienten zeichnen sich durch Multimorbidität, Polypharmazie, Adipositas, Immobilität, Pre‑/Frailty und Vulnerabilität für postoperative Komplikationen aus.
- Geriatrische Expertise und Optimierung modifizierbarer Risikofaktoren sollten in der elektiven Orthopädie zukünftig routinemäßig zum Einsatz kommen.
- Orthogeriatrische Co-Management-Modelle können womöglich Komplikationen reduzieren und werden aktuell in Deutschland wissenschaftlich evaluiert.

## Supplementary Information

Ausführliche Erläuterung der Short Physical Performance Battery (SPPB) sowie der Frailty-Kriterien nach Fried
