# Supplementary material for: Risk and complication profiles of orthogeriatric patients in elective hip and knee joint replacement
Source: Z Gerontol Geriatr. 2024 Apr 19;58(2):115–22. [Article in German] doi: 10.1007/s00391-024-02295-3 (PMC11870982; doi:10.1007/s00391-024-02295-3)
Supplement: Supplementary file 1 — Ausführliche Erläuterung der Short Physical Performance Battery (SPPB) sowie der Frailty-Kriterien nach Fried [file 391_2024_2295_MOESM1_ESM.docx]

**Supplement**

**e1:**

**Short Physical Performance Battery (SPPB)**

Die SPPB ist eine Testbatterie, welche die motorischen Kriterien Gleichgewicht, Ganggeschwindigkeit und Muskelkraft vereint. Sie beinhaltet die progressive Testung der statischen Balance (Side-by-Side-, Semi-Tandem- und Tandem-Stand), die Messung der Ganggeschwindigkeit über 4 m und den Chair Rise Test (5-mal vom Stuhl aufstehen und hinsetzen). Der Test ermöglicht sowohl einen Summenscore als auch einzelne Werte in den drei motorischen Dimensionen. Maximal sind 12 Punkte im Gesamtscore erreichbar [1].

**Frailty-Kriterien nach Fried**

Die Erfassung der Gebrechlichkeit (Frailty) nach Fried et al. [2] erfolgt anhand von 5 Kriterien:

- Unbeabsichtigter Gewichtsverlust von > 5 kg/Jahr
- Selbstberichtete körperliche und geistige Erschöpfung
- Reduzierte Ganggeschwindigkeit über 5 m
- Muskuläre Schwäche gemessen per Handkraft mittels Jamar® Dynamometer
- Reduzierte körperliche Aktivität (Aktivitätslevel nach Swiss Health Observatory)

Jedes Kriterium wird mit 0 oder 1 bewertet. Daraus ergibt sich ein Gesamtscore von 0-5.

0 Kriterien bedeutet robust, 1 oder 2 Kriterien stehen für pre-frail und ≥ 3 Kriterien sprechen für Frailty.

1. Guralnik JM, Simonsick EM, Ferrucci L et al (1994) A short physical performance battery assessing lower extremity function: association with self-reported disability and prediction of mortality and nursing home admission. J Gerontol 49:M85-94
2. Fried LP, Tangen CM, Walston J et al (2001) Cardiovascular Health Study Collaborative Research Group. Frailty in older adults: evidence for a phenotype. J Gerontol A Biol Sci Med Sci 56(3):M146-56
